# Synthesis of Macroporous Polystyrene by the Polymerization of Foamed Emulsions**

**DOI:** 10.1002/anie.201107806

**Published:** 2012-01-20

**Authors:** Fabian Schüler, Debora Schamel, Anniina Salonen, Wiebke Drenckhan, Michael D Gilchrist, Cosima Stubenrauch

**Affiliations:** * Institut für Physikalische Chemie, Universität StuttgartPfaffenwaldring 55, 70569 Stuttgart (Germany); School of Mechanical and Materials Engineering, University College DublinBelfield, Dublin 4 (Ireland); Laboratoire de Physique des Solides, UMR 8502, Université Paris-SudBâtiment 510, 91405 Orsay Cedex (France)

**Keywords:** foamed emulsions, foams, macroporous polymers, UV polymerization

Polymer foams are of great commercial interest and are used in diverse fields such as packaging, insulation, and impact protection.^1^ Depending on the application, a foam must meet specific requirements. Thus great effort has been invested in the determination and manipulation of foam properties. Material composition and cellular structure constitute crucial parameters when it comes to the tailoring of foams. As conventional manufacturing, where foams are produced from polymer melts and blowing agents, is a very complex process, it is hard to control the product’s morphology and properties.

In recent years alternative methods for the synthesis of polymer foams have been proposed which make use of templates: a template is generated first and the actual polymer is subsequently synthesized. For example, emulsions have been found to be suitable templates for the synthesis of porous materials. In particular water-in-oil emulsions with a high concentration of the dispersed phase (high-internal-phase emulsions, HIPEs) have attracted a great deal of attention.^2^ These systems consist of a polymerizable continuous phase and a dispersed phase, which is removed after the polymerization. As most monomers relevant for polymer foam production are hydrophobic, studies usually deal with the polymerization of water-in-oil emulsions, but there are also some examples where oil-in-water emulsions have been polymerized.^3^ It has been shown that by varying the system parameters the foam morphology and mechanical behavior can be fine-tuned.^4^ New materials were obtained by subsequent processing of the porous polymers,^5^ and recently particle-stabilized emulsions (Pickering emulsions) were successfully applied for the synthesis of various nanocomposites.^6^

The discovery that particles can also attach to gas–liquid interfaces and stabilize air bubbles^7^ has laid the foundations for another practical route towards the controlled synthesis of porous materials from templates.^8^ The potential of this approach for the synthesis of different porous polymers has been shown recently.^9^ A similarly successful approach is to directly generate perfectly monodisperse and highly ordered polymer foams by microfluidic flow focussing techniques.^10^

We describe herein a novel concept for the synthesis of macroporous polystyrene by the UV-initiated photopolymerization of foamed oil-in-water emulsions. So far, there have been only a few studies on foamed emulsions,^11^ and, to the best of our knowledge, their use in the synthesis of polymer foams has not been reported yet. In our studies we focused on styrene-based foamed emulsions which are polymerized by UV irradiation; however, we believe that this approach can be applied to a much wider range of monomers and polymerization routes. The route we worked out consists of three principal steps. In the first step, a stable oil-in-water (here: styrene-in-water) emulsion is formulated. Next, the emulsion is foamed by bubbling N_2_ through the sample. Both the emulsion and the foamed emulsion are stabilized by the same surfactant; we found the anionic sodium dodecyl sulfate (SDS) to have the best performance. Finally, the resulting foamed emulsion is polymerized by exposure to UV light (Figure 1).

**Figure 1:**
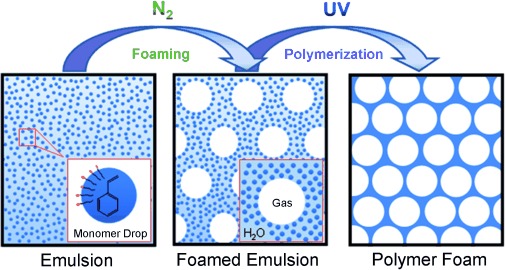
Synthesis of macroporous polymers by foaming monomer-containing emulsions and subsequent polymerization.

The emulsions were prepared by consecutive addition of styrene, water, glycerol (some samples were prepared without glycerol), and SDS. We homogenized the mixtures by ultrasonication, which led to small and relatively uniform droplets 0.5–1 μm in diameter.

At the outset we conducted extensive foaming experiments with mixtures stabilized by different surfactants at various styrene-to-water ratios. Emulsions stabilized by SDS turned out to foam significantly better than those prepared with a variety of non-ionic surfactants. As all SDS-containing emulsions were stable for several hours, we expected them to also be stable during foam generation. A composition of 65 vol % styrene and 35 vol % hydrophilic phase (water or water+glycerol) was found to be the best compromise regarding good foamability and sufficient foam lifetime. Note that the maximum density of a random close packing of spheres is 64 vol %. Thus at 65 vol % styrene the emulsion droplets start to jam, which renders foaming more difficult but at the same time enhances the foam lifetime once the foam has been generated.11a, 12 Figure 2 a shows an example of a single foam bubble surrounded by closely packed emulsion droplets.

**Figure 2:**
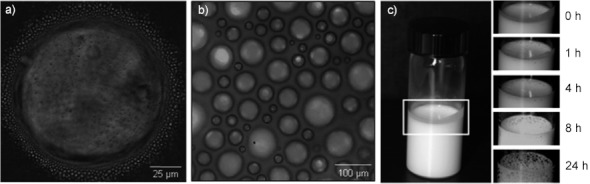
a, b) Optical light microscopy pictures of foamed emulsions. c) Evaluation of foam lifetime for a foamed emulsion containing 5 wt % SDS and 15 vol % glycerol. Foam lifetime is defined as the time after which significant collapsing of bubbles appeared (this can be seen on the picture taken after 8 h). Pictures were taken with the optimal system (see text for details).

Further optimization was achieved by adjusting the foam generation process and varying the composition of the emulsion. With regard to the first parameter, we found that whipping the emulsions with a mechanical stirrer at mixing speeds of 1600 rpm and a mixing time of 4 min leads to sufficiently stable foamed emulsions with appropriate foam densities and bubble sizes (see the Supporting Information). Figure 2 b displays a typical image of a generated foamed emulsion. The rather broad distribution of bubble sizes is due to the applied foaming technique.^13^ Figure 2 c illustrates the time-dependent changes in the structure of foamed emulsion. In the following we define “foam lifetime” as the time after which significant collapsing of foam bubbles appears (as seen in the picture taken after 8 h).

xxAfter having optimized the foam generation process we adjusted the composition of the emulsion in order to optimize the foam lifetime. (Note that we did not optimize the process in an iterative way although changes in the composition may require an adjustment of the foam generation process and vice versa.) Gas contents were calculated comparing the volume of the nonfoamed emulsion and the volume of the foam generated from it. All foamed emulsions were examined by light microscopy, and average bubble sizes were obtained by evaluating the respective images (see Supporting Information).

We first tested the influence of the SDS concentration on the stability of the foamed emulsions. The stability of a foam in the presence of oil droplets is critically governed by the stability of the so-called pseudo-emulsion film which forms between the air/water interface and an oil drop.11b If this film is stable, the oil drops do not enter the air/water interface but accumulate in the plateau borders of the foam, thus slowing down liquid drainage.^11^ Since the amount of added SDS must be shared between the gas/liquid and liquid/liquid interfaces to stabilize both foam and emulsion, surfactant concentrations much higher than the critical micelle concentration (cmc) were added to the mixture (the cmc for SDS in pure water at room temperature is roughly 0.26 wt %). All emulsions with total SDS concentrations equal to or exceeding 1 wt %, which corresponds to 2.9 wt % in the aqueous phase, yielded stable foams.

Figure 3 shows a decrease of the bubble size and an increase of foam lifetime with increasing SDS concentration. Gas contents up to 82 % were achieved, but foams strongly degraded within 2 h, even at the highest surfactant concentrations. Average bubble diameters were between 70 and 90 μm with broad size distributions. With regard to the optimal SDS concentration, we needed to find a compromise between foam lifetime and solubility: On the one hand, the foam properties did not change significantly upon an increase of the SDS concentration from 5 to 7 wt %. On the other hand, the solubility of SDS in pure water at room temperature is approximately 15 wt %.^14^ Thus a total SDS concentration of 5 wt %, which corresponds to 14.3 wt % in the aqueous phase, was chosen for all further experiments in order to ensure good solubility.

**Figure 3:**
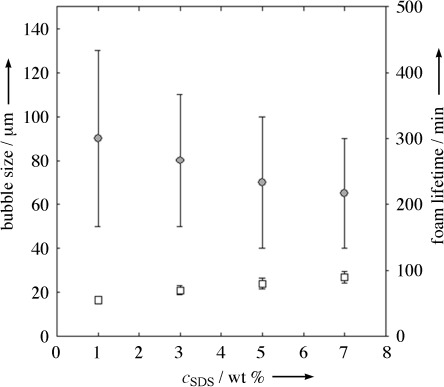
Dependence of bubble diameter (○) and foam lifetime (□) on SDS concentration based on the total sample mass. The emulsions contained 65 vol % styrene and 35 vol % water.

The next step was to vary the composition of the continuous phase by partially replacing water with glycerol. As glycerol has a much higher viscosity than water, it slows down the creaming of the emulsions and the drainage of the foamed emulsions.11c We varied the glycerol concentration (*c*_glycerol_) while maintainin the total amount of the continuous phase (35 vol %). These emulsions were then foamed and we found that the viscosity did not significantly affect the final foam density. However, foam bubble sizes and bubble size dispersities decreased with increasing *c*_glycerol_ as is shown in Figure 4. This observation is due to the fact that our foaming technique is based on the application of shear forces to air/fluid interfaces, which are inversely proportional to the viscosity of the fluid.^15^ Most importantly, the stability of the foamed emulsion increases significantly with increasing *c*_glycerol_ and thus increasing viscosity of the continuous phase: foams with *c*_glycerol_=15 vol % were completely stable for almost 7 h. There are two reasons for this tremendous increase of stability. Firstly, it can be attributed to the smaller and more uniform foam bubbles (less Ostwald ripening). Secondly, the increased viscosity of the emulsion significantly slows down the gravity-driven drainage. Because of solubility problems, the highest *c*_glycerol_ tested was 15 vol %.

**Figure 4:**
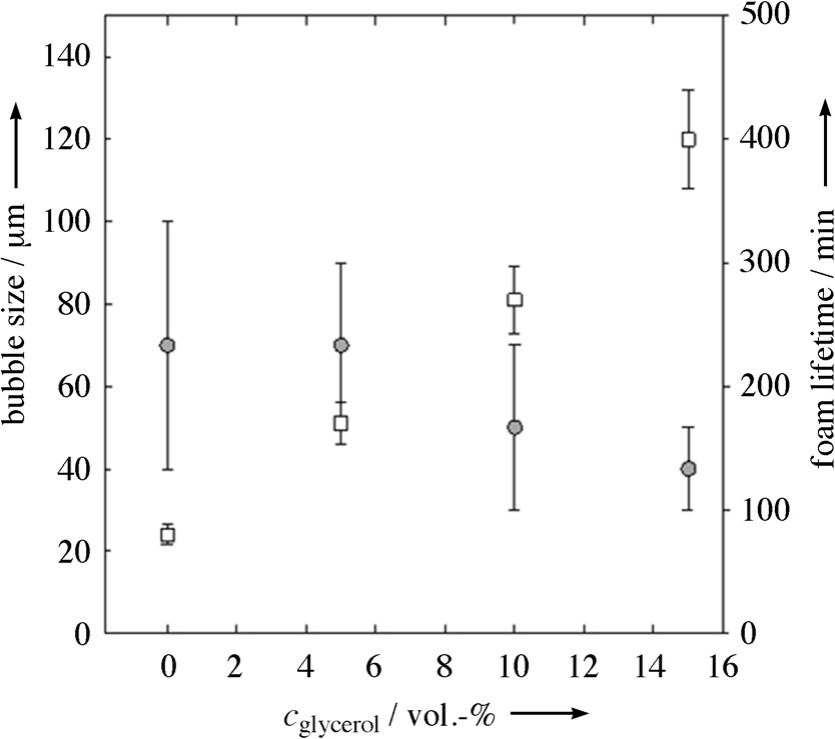
Dependence of bubble diameter (○) and foam lifetime (□) on the glycerol concentration based on the total sample mass (*c*_glycerol_=15 vol % means 15 vol % glycerol and 20 vol % water in the total system). The emulsions contained 65 vol % styrene, 35 vol % continuous phase with different amounts of glycerol and 5 wt % SDS.

As a result of the prescreening tests the optimal composition of the emulsion was chosen to be 65 vol % styrene, 20 vol % water, 15 vol % glycerol, and 5 wt % SDS. Because of their higher stability during the polymerization, liquid foams with gas contents of only 60–70 % were prepared. These optimized foamed emulsions were then polymerized by exposure to UV light.

For the photopolymerization we tested different photoinitiators, which were mixed with the emulsions before foaming. We tested dimethyl benzyl ketal (DBK), benzoin (BZ), diphenylacetone (DPA), and diphenyl(2,4,6-trimethylbenzoyl)phosphine oxide (TPO), each at a concentration of 2 wt % relative to the overall sample mass. In order to limit the heating of the sample, which destabilizes the foam, the lamps’ infrared radiation was extinguished by an optical filter. We found that an irradiation time of 2 h sufficed to convert the foamed emulsion into polymeric material. The samples were then dried and purified by Soxhlet extraction. Table 1 lists the results from the polymerization experiments with different photoinitiators.

**Table 1:** Results from polymerizing foamed emulsions with different initiators at 2 wt %.[a]

Initiator	Effect of polymerization on foam structure	*M*_n_ (GPC RI/UV) [g mol^−1^][b]	*PD* (GPC RI/UV)[b]

DBK	macroporosity retained	21 000/20 000	1.8/1.8
BZ	macroporosity retained	25 000/26 000	3.7/3.3
TPO	macroporosity retained	53 000/44 000	2.2/3.1
DPA	structure collapses	25 000/25 000	3.6/3.9

[a] Molecular weights Mn and polydispersities PD were obtained from GPC measurements.

[b] RI=refractive index detector, UV=UV light detector.

Gel permeation chromatography (GPC) measurements were performed after Soxhlet treatment of the samples, and molecular weights between 20 000 and 53 000 g mol^−1^ and polydispersities between 1.8 and 3.9 were obtained. It is generally known that higher molecular weights improve the mechanical performance of polymers; hence the molecular weight needs to be increased. All initiators apart from DPA gave macroporous polymers with negligible shrinkage upon solidification. On the other hand, the use of DPA led to a complete collapse of the foam. The consistency of the collapsed material was more slushy than solid, most likely because of low monomer conversion. The analysis of the purified residue indicated the formation of polystyrene with a broad polydispersity.

As the highest molecular weights were obtained with the initiator TPO, the subsequent experiments and analyses were conducted with this foam. We found that sintering of the polymer material is required to obtain a smooth surface. Thus differential scanning calorimetry (DSC) and thermal gravimetric analysis (TGA) measurements were performed to determine suitable conditions for the subsequent thermal treatment of the polymer. It was found that the decomposition of our best polymer foam started at 295 °C, while its glass transition was around 100 °C. Thus sintering was performed at a temperature close to *T*_g_, namely at 110–120 °C, for 3 h. The difficulty of this step consisted in achieving a polymer with a homogeneous surface without causing structure collapse. After the thermal processing, the sample thickness was measured and compared to the initial thickness of the liquid foam. The best batches displayed shrinkages of not more than 20 %. The macroporous polymers were further analyzed by scanning electron microscopy (SEM; see Figure 5). The image shows a continuous macroporous structure with closely packed cells and some openings (so-called windows2d) between adjacent pores. Cracks and scratches in the polystyrene bulk material were eliminated by sintering (see the Supporting Information). We find that the structure of the foamed emulsion—the template—could be transferred to the solid macroporous polystyrene without significant changes. To quantify this, the liquid template and the final polymer foam are compared in Table 2. The density was calculated by weighing specimens of known volume, and the gas content was obtained from the density ratio of macroporous polystyrene and bulk polystyrene (see the Supporting Information).

**Figure 5:**
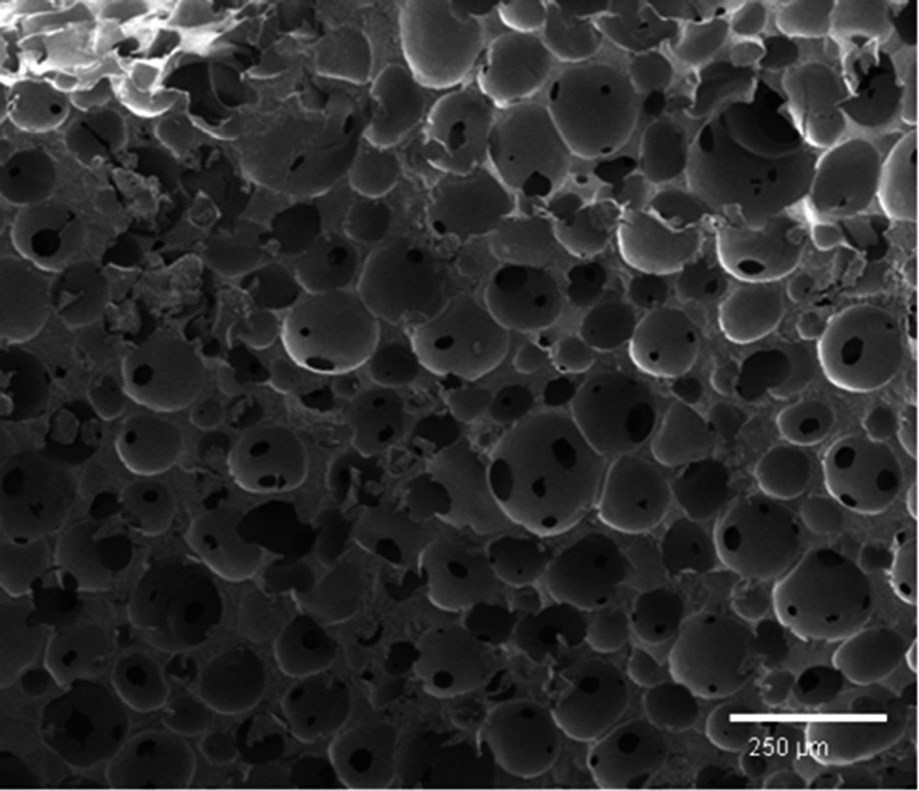
SEM images of polystyrene foams obtained by polymerizing foamed emulsions with the UV initiator TPO.

**Table 2:** Properties of the foamed emulsion (the template) and the final macroporous polystyrene.

	Gas content [%]	Density [g cm^−3^]	Average bubble/pore size [μm]

foamed emulsion	64±5	0.37±0.05	46±12
polymer foam	78±4	0.24±0.04	76±30

As can be seen in Table 2, the gas content of the polymer foam is slightly higher than that of the foamed emulsion, which most likely is due to the removal of the emulsions’ hydrophilic phase. The assumption that all emulsion components except the polymerized monomer can be removed from the final product leads to a final gas content of 77 %, which is very close to our results. Moreover, as can be seen in Figure 6, the pore size distribution of the polymer foam is broader than that of the liquid template and the maximum is shifted to higher values, which is probably due to bubble coalescence during the polymerization.

**Figure 6:**
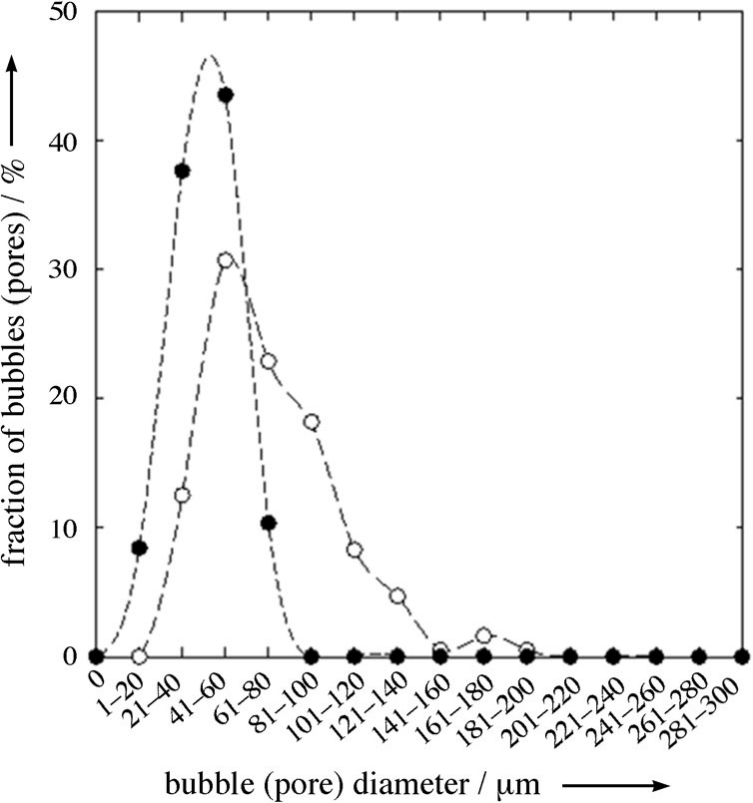
Bubble (•) and pore size distributions (○) for liquid foamed emulsions and macroporous polystyrene.

The average diameters of the windows were calculated to be (30±10) μm. Windows are commonly found in other macroporous polymers that are synthesized from HIPEs. Generally their formation is attributed to the destabilization of the emulsion films upon solidification, but the detailed mechanism of their formation is not yet fully understood.^16^ In our case we believe that the window-forming mechanism is already initiated in the liquid state. In some places, the small styrene droplets are expelled from the thin aqueous film separating two bubbles—as is commonly seen in foamed emulsions.11f Upon polymerization, those films do not solidify and thus create highly spherical holes. As a result, our approach allows the generation of polymer foams with a nonnegligible fraction of interconnected pores. While the high density and strong connectivity provides mechanical stability, the presence of the windows allows air, fluids, or other materials to penetrate the material. Control over this balance is searched for a wide range of applications, including solid supports, filtration materials, and bio-inspired scaffold structures.2d, 17

In summary we have presented a simple and versatile route for the creation of polystyrene foams from foamed emulsions, which is a promising alternative to other methods that make use of templates for material production. The simplicity of this approach makes any type of foaming method possible, thus offering a tunability of the bubble size and structure of the liquid foam. During polymerization and subsequent processing, the structure of the foamed emulsion was retained and the samples shrank very little. In other words, the use of a foamed emulsion as a template allows the manufacturing of specimens with controlled structural properties. Owing to its generality the strategy proposed by us can be extended to a wide range of other monomers and composites which can be polymerized from emulsions. Future goals include the increase of the molecular weight, a more detailed study of the structure of both the template and the polymer foam, and an extension of the concept to other polymers.

## Supporting information

Detailed facts of importance to specialist readers are published as ”Supporting Information”. Such documents are peer-reviewed, but not copy-edited or typeset. They are made available as submitted by the authors.
