# Supplementary material for: Synthesis of Macroporous Polystyrene by the Polymerization of Foamed Emulsions
Source: Angew Chem Int Ed Engl. 2012 Jan 20;51(9):2213–7. doi: 10.1002/anie.201107806 (PMC3415665; doi:10.1002/anie.201107806)
Supplement: Supplementary file 1 [file anie0051-2213-SD1.pdf]

Angewandte Chemie
Eine Zeitschrift der Gesellschaft Deutscher Chemiker

Supporting Information
© Wiley-VCH 2012
69451 Weinheim, Germany

Synthesis of Macroporous Polystyrene by the Polymerization of Foamed Emulsions**

*Fabian Schüler, Debora Schamel, Anniina Salonen, Wiebke Drenckhan, Michael D. Gilchrist, and Cosima Stubenrauch**

anie_201107806_sm_miscellaneous_information.pdf

Supporting Information

1. Materials

SDS (sodium dodecyl sulfate, ≥99 %), DBK (dimethyl benzyl ketal, 99 %), BZ (benzoin, 99.5 %), DPA (diphenylacetone, 99 %), TPO (diphenyl-(2,4,6-trimethylbenzoyl)-phosphine oxide, 97 %) and glycerol (≥99 %) were supplied from Sigma Aldrich and used as received. Styrene (99 %) was purchased from VWR and purified by vacuum distillation when used for polymerization. For the foaming tests styrene was used as received. Distilled water was degassed by flushing with N_2 for 20 min. Plantacare surfactants were supplied from Cognis (now part of BASF) and their specifications are listed in Table .

Table S1: Plantacare surfactants used for foaming (C = alkyl chain; G = glucose)

Surfactant	Chemical formula	Molecular weight [g/mol]	Active matter [%]
Plantacare 810 UP	$C_{8-10}G_{1.5}$ (~C_{9-10})	370	60
Plantacare 818 UP	$C_{8-16}G_{1.5}$ (~C_{11-12})	398	50
Plantacare 2000 UP	$C_{8-16}G_{1.5}$ (~C_{12})	398	50

2. Preparation and Foaming of Emulsions

For the generation of the emulsion the compounds were added in the following order: styrene, water, glycerol (if required), SDS. The concentration of the liquids is given in volume percentage (calculated from the respective densities of styrene, water, and glycerol), whereas all amounts of solid compounds are given in weight percentage (related to the sample´s total mass). The mixtures were stirred for 30-40 minutes, then the photoinitiator was added and stirring was continued for further 20-30 minutes (only for polymerizations, not for liquid foam optimization experiments). The samples were then treated with a *Bandelin HD-2200* ultrasound homogenizer for 40 s at a device power of 30%. For foaming, a small amount of the emulsions (typically ~2 ml) was placed in a vial and mechanically whipped with a KPG stirrer under N_2 atmosphere. The mixing shaft was taken from a conventional milk frother. The bubble size and thus the gas content were varied via the mixing speed and the mixing time. Optimal conditions were found at mixing speeds of 1600 rpm and mixing times of 4 minutes. The results are shown in Figure S1 and S2.

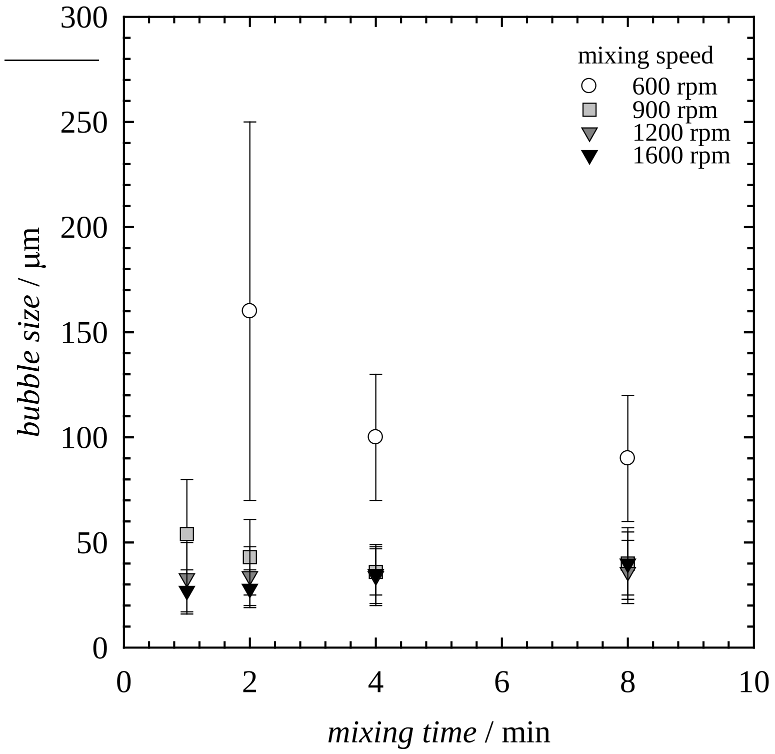

Figure S1: Dependence of the bubble size of the foamed emulsions on mixing speed and mixing time.

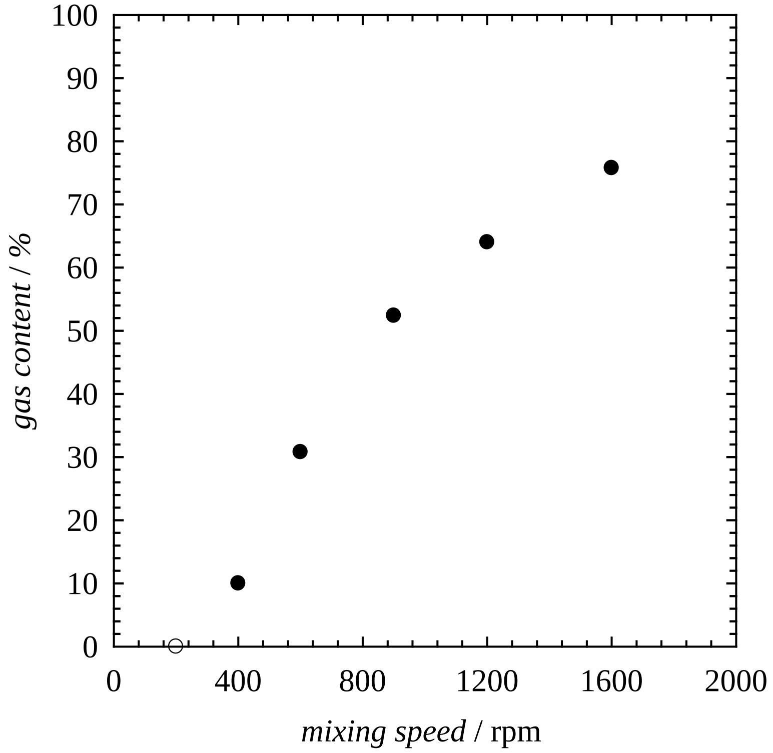

Figure S2: Dependence of the gas content of the foamed emulsion on mixing speed at a fixed mixing time of 4 min.

3. Characterization of the Emulsions

Microscopy pictures of the emulsions were taken with an Olympus BX 51 microscope. At least 100 droplets were evaluated with the software Image J (http://rsbweb.nih.gov/ij/) and an average droplet size as well as a standard deviation was calculated.

4. Characterization of the Liquid Foams

Microscopy pictures of the liquid monomer foams were taken with an Olympus BX 51 microscope. At least 50 foam bubbles were evaluated with the software Image J (http://rsbweb.nih.gov/ij/) and an average droplet size as well as a standard deviation was calculated. The stabilities of the generated foams were estimated by measuring the time after which a serious collapse of the foam is observed. An error of 10 % was assumed. The gas content was estimated by measuring of the height difference between the initial emulsion and the freshly generated monomer foam according to

$$\text{gas content} = \frac{h_{Foam} - h_{Emulsion}}{h_{Foam}} \cdot 100$$

$$\Delta\text{gas content} = \left|\frac{\partial gas\ content}{\partial h_{foam}}\right| \cdot \Delta h_{foam} + \left|\frac{\partial gas\ content}{\partial h_{emulsion}}\right| \cdot \Delta h_{emulsion}$$

$$= \left(\left|-\frac{h_{emulsion}}{h_{foam}^2}\right| \cdot \Delta h_{foam} + \left|\frac{1}{h_{foam}}\right| \cdot \Delta h_{emulsion}\right) \cdot 100$$

An error of 0.1 cm was assumed for the heights of both foam and emulsion due to inaccuracy of the measurement.

5. Characterization of the Polymer Foams

(a) Molecular weights and polydispersity indices (PDIs) of the polymers were determined by gel permeation chromatography (GPC). The measurements were carried out at 30°C on Polymer Laboratories columns (PLgel 10 mm MIXED-B, 7.5x300 mm) using a Waters Autosampler 2707, a Waters 2489 UV spectrometer detector (254 nm), and a Waters 2414 refractive index detector. For the measurements 2-3 mg of the polymer sample was dissolved in 2 ml of chloroform. The GPC measurements were performed with assistance of Dr. Dongren Wang from the group of Prof. Dr. Michael Buchmeiser at the Institute of Polymer Chemistry at Stuttgart University.

(b) Differential Scanning Calorimetry (DSC) was carried out at on a Perkin Elmer Pyris 1. The following temperature program was used:

1) Hold temperature at 20 °C for 5 min
2) Heat from 20 °C to 150 °C at 10 °C/min
3) Cool from 150 °C to 20 °C at 10 °C/min
4) Heat from 150 °C to 20 °C at 10 °C/min

Glass transition temperatures were estimated from the second heating curve.

(c) Thermal Gravimetric Analysis (TGA) measurements were conducted with a Netzsch STA 409. Measurements were carried out under air atmosphere.

(d) Scanning Electron Microscopy (SEM) pictures were taken with a FEI Quanta 3D electron microscope. Samples were sputter-coated with gold nanoparticles to increase charge dissipation.

(e) The polymer foam thickness was measured at different regions of the sample with a caliper. From these measurements the polymer volume could be calculated with an accuracy of ±0.2 cm for each dimension. Polymer densities were estimated by weighting specimen of known volume. Shrinkage due to polymerization and sintering, respectively, was calculated according to

$$\text{shrinkage} = 1 - \frac{V_{PS\,Foam}^{Final}}{V_{PS\,Foam}^{Initial}} \cdot 100$$

$$\Delta\text{shrinkage} = \left|\frac{\partial shrinkage}{\partial V_{PSfoam}^{Final}}\right| \cdot \Delta V_{PSfoam}^{Final} + \left|\frac{\partial shrinkage}{\partial V_{PSfoam}^{Initial}}\right| \cdot \Delta V_{PSfoam}^{Initial}$$

$$= \left(\frac{1}{\partial V_{PSfoam}^{Initial}} \cdot \Delta V_{PSfoam}^{Final} + \frac{\partial V_{PSfoam}^{Final}}{\partial V_{PSfoam}^{Initial\,2}} \cdot \Delta V_{PSfoam}^{Initial}\right) \cdot 100$$

The gas content of the polymer samples was calculated via the density ratio of the polymer foam and pure polystyrene (ρ = 1.05 g/cm^3) according to

$$\text{gas content} = 1 - \frac{\rho_{PS\,Foam}}{\rho_{PS\,Bulk}} \cdot 100$$

$$\Delta\text{gas content} = \left|\frac{\partial gascontent}{\partial \rho}\right| \cdot \Delta\rho = \frac{1}{1.05} \cdot \Delta\rho_{PSfoam} \cdot 100$$

6. Influence of Emulsion Composition on Foaming Experiments

Emulsion preparation and foaming were conducted as described before. All weight and volume indications are related to the total sample weight or volume. The following tables list all experiments and the results.

(a) Variation of surfactant type

Initial weights: Styrene (5.90 g; 65 vol.-%), water (3.50 g; 35 vol.-%), surfactant (see table; since the Plantacare surfactants are of technical grade with an active matter less than 100%, the equivalent molar amount to SDS was used).

Table S2: Foaming experiments with different surfactants

Surfactant	Active matter [%]	Initial weight [g]	Active amount [mmol]	Active amount [wt.-%]	Foam stability [min]
Plantacare 810 UP	60	1.06	1.73	10.1	80±8
Plantacare 818 UP	50	1.38	1.73	12.8	60±6
Plantacare 2000 UP	50	1.38	1.73	12.8	60±6
SDS	100	0.50	1.73	5.1	80±8

(b) Variation of surfactant concentration

Initial weights: Styrene (5.90 g; 65 vol.-%), water (3.50 g; 35 vol.-%), SDS (see table)

Table S3: Foaming experiments at different surfactant concentrations

SDS concentration [g/wt.-%]	Emulsion droplet size [µm]	Gas content [%]	Foam stability [min]	Foam bubble size [µm]
0.10/1	0.6±0.1	80±3	55±6	90±40
0.30/3	0.6±0.1	73±4	70±7	80±30
0.50/5	0.6±0.1	77±4	80±8	70±30
0.71/7	0.6±0.1	77±4	90±9	65±25

(c) Variation of glycerol content in the aqueous phase

Initial weights: Styrene (5.90 g; 65 vol.-%), SDS (0.50 g; 5 wt.-%), glycerol + water (see table)

Table S4: Foaming experiments at different glycerol contents in the aqueous phase

Glycerol concentration [g/vol.-%]	Water concentration [g/vol.-%]	Emulsion droplet size [µm]	Gas content [%]	Foam stability [min]	Foam bubble size [µm]
0.00/0	3.50/35	0.6±0.1	77±4	80±8	70±30
0.65/5	3.00/30	0.5±0.1	81±4	170±17	70±20
1.25/10	2.50/25	0.5±0.1	82±4	270±27	50±20
1.90/15	2.00/20	0.5±0.1	72±4	400±40	40±10

(d) Variation of hydrophobic/hydrophilic ratio

The ratio between water and glycerol was kept constant. The sample with 74 vol.-% styrene contained less surfactant compared to the others due to the solubility limit of SDS.

Table S5: Composition of samples with different contents of styrene

Ratio hydrophobic/hydrophilic	Styrene [g/vol.-%]	Water [g/vol.-%]	Glycerol [g/vol.-%]	SDS [g/wt.-%]
50/50	4.55/50	2.87/29	2.71/21	0.53/5
65/35	5.90/65	2.00/20	1.90/15	0.52/5
74/26	6.73/74	1.49/15	1.40/11	0.40/4

Table S6: Foaming experiments with the samples listed in Table S3

Ratio hydrophobic/hydrophilic	Gas content [%]	Foam stability [min]
50/50	80±4	150±15
65/35	80±5	400±40
74/26	35±2	80±8

7. Polymerization and subsequent processing of foamed emulsions

Foams were prepared as explained above and filled into a water-cooled aluminum frame (dimensions 3cm x 4cm x 0.3cm). The bottom consisted of UV light-transmissive glass which was covered with UV light-transmissive PTFE-film. The sample consisted of styrene (5.9 g; 65 vol.-%), water (2.00 g; 20 vol.-%), glycerol (1.90 g; 15 vol.-%), SDS (0.50 g; 5 wt.-%), and photoinitiator (0.21 g; 2 wt.-%). Note that we tested the influence of the initiator concentration on the polymerization process. It was found that for initiator concentrations lower than 2 wt.-% (related to the sample’s total mass) the foam collapses during irradiation with UV light. Although we have not yet an explanation for this observation, the high initiator concentration is certainly one reason for the (too) low molecular weight of the resulting polymer.

Polymerization was initiated with two MH 250 W UV lamps, which were placed 20 cm above and below the frame. Optical filters (Schott UG 11) were placed between the UV source and the foam to avoid heat exposure of the sample. Samples were irradiated for 120 min in total, irradiation was conducted from the top only for the first 70 minutes and then from both sides in order to prevent foam collapse. After the polymerization, the samples were dried overnight at 70 °C. Purification of the resulting polymer was achieved *via* Soxhlet extraction in ethanol for 3 hours. Sintering was conducted in an oven between 110 °C and 120 °C for 3-4 hours.

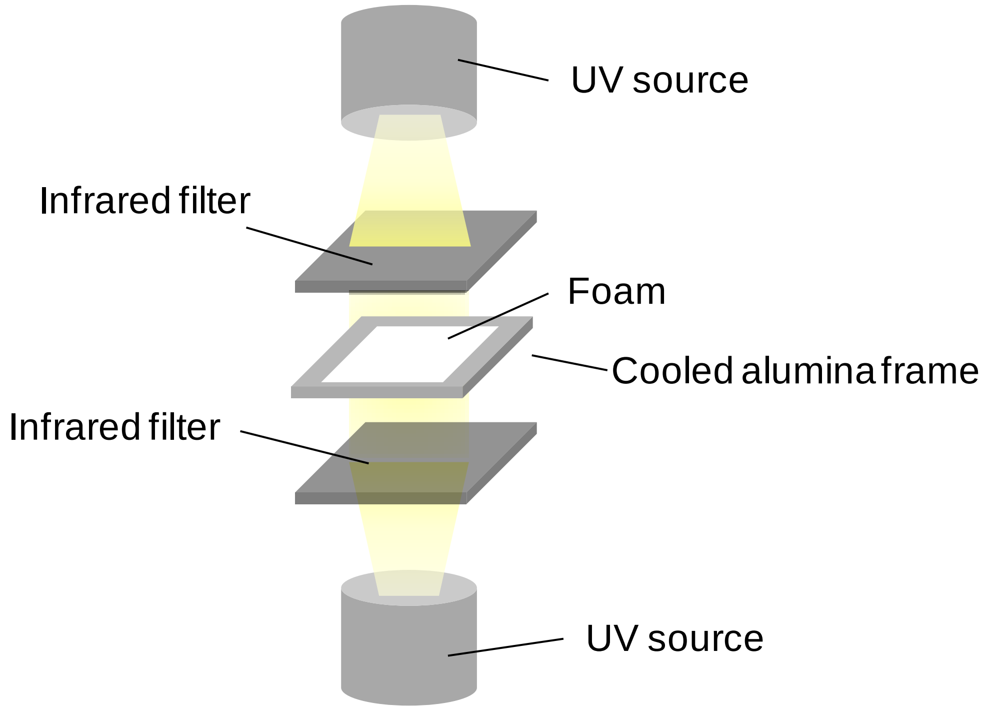

Figure S3: Schematic picture of the polymerization setup

8. SEM pictures for analysis of sintering process

SEM pictures of foams after each processing step were analyzed in order to evaluate the suitability of the applied conditions.

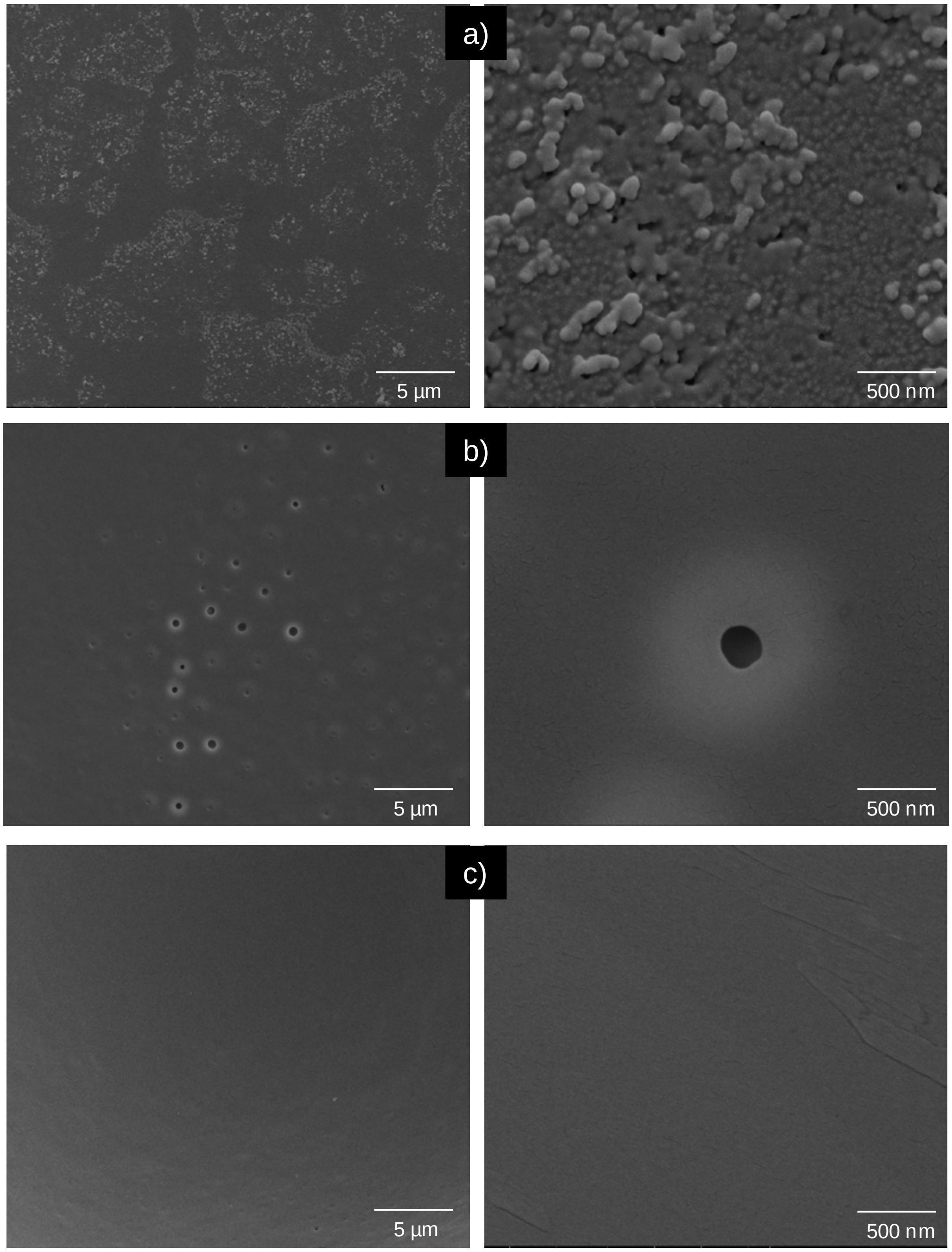

Figure S4: SEM close-ups of macroporous polystyrene after different processing steps: (a) after polymerization and drying, (b) after Soxhlet extraction with ethanol, and (c) after sintering at 110 °C. Two different magnifications are shown.
